# Evolutionary stability and the rarity of grandmothering

**DOI:** 10.1002/ece3.2958

**Published:** 2017-04-10

**Authors:** Jared M. Field, Michael B. Bonsall

**Affiliations:** ^1^ Wolfson Centre for Mathematical BiologyMathematical InstituteUniversity of OxfordOxfordUK; ^2^ Mathematical Ecology Research GroupDepartment of ZoologyUniversity of OxfordOxfordUK

**Keywords:** evolutionary game theory, grandmother hypothesis, grandparent–grandoffspring conflict, mathematical ecology

## Abstract

The provision of intergenerational care, via the Grandmother Hypothesis, has been implicated in the evolution of postfertile longevity, particularly in humans. However, if grandmothering does provide fitness benefits, a key question is why has it evolved so infrequently? We investigate this question with a combination of life‐history and evolutionary game theory. We derive simple eligibility and stability thresholds, both of which must be satisfied if intergenerational care is first to evolve and then to persist in a population. As one threshold becomes easier to fulfill, the other becomes more difficult, revealing a conflict between the two. As such, we suggest that, in fact, we should expect the evolution of grandmothering to be rare.

## 1 Introduction

Data on historical agricultural populations and modern hunter‐gatherers show that these groups enjoy significant postfertile periods (Alberts et al., 2013; Blurton Jones, Hawkes, & O'Connell, 2002; Gurven & Kaplan, 2007; Levitis, Burger, & Lackey, 2013). Modern medicine cannot then fully explain the life‐history oddity of increased longevity with reproductive inactivity.

Taking an evolutionary approach, the Grandmother Hypothesis instead proposes that this reproductive inactivity is in fact adaptive (Hawkes, OConnell, Jones, Alvarez, & Charnov, 1998). With the sacrifice of continued reproduction, an individual may increase their inclusive fitness by decreasing the interbirth intervals of their offspring. The care that would otherwise be put into one's own children can now be put into weaned (and increasingly independent) grandchildren, allowing their own offspring to reproduce again sooner. Otherwise put, the cost of a reduced relatedness coefficient may be outweighed by an increase in total number of grandchildren resulting from the diverted care. Several models have now shown how such a benefit could be realized (Chan, Hawkes, & Kim, 2016; Kim, Coxworth, & Hawkes, 2012; Kim, McQueen, Coxworth, & Hawkes, 2014). In this way, a causal connection is made between the provision of intergenerational care and human postfertile longevity.

A valid objection to the Grandmother Hypothesis, however, is if grandmothering can result in a higher fitness, why are significant postfertile life stages so rare? Among vertebrates in the wild, only humans, *Globicephala macrorhynchus* (pilot whales) and *Orcinus orca* (resident killer whales), have a significant proportion of individuals with such a life history (Croft, Brent, Franks, & Cant, 2015). In this study, we present a model to investigate this objection. Our model assumes only that individuals transition through various life stages and that there is an average time to conception and gestation. In one of those stages, individuals have the option to provide care for a certain number of their grandchildren thereby allowing their own offspring to reproduce again sooner.

By comparing inclusive fitnesses of individuals that provide intergenerational care with those that instead continue to reproduce into old age, we arrive at a necessary condition for grandmothering to be an evolutionarily stable strategy (ESS). This condition, or stability threshold, relates the number of grandchildren that care must be given to with the ratio of the length of the first two life stages. It tells us nothing about when or how grandmothering may arise initially in a population, but places restrictions on when it will persist.

We then make the observation that if a grandmother is to provide care for even one set of grandchildren, their expected postfertile stage must be sufficiently long. More precisely, for grandmothering to be adaptive, it must be the case that postfertile life exceeds the time taken to raise a weaned child to independence. If this were not the case, grandmothers would not be able to shorten their offspring's time between births by caring for some infants themselves. In this way, we derive an eligibility threshold that tells us when grandmothering is a strategy with any possible evolutionary advantage. These eligibility and stability criteria must both be satisfied for grandmothering to evolve and then, most importantly for our purposes, to persist.

Our analyses show that there is conflict between the stability and eligibility thresholds. As it becomes increasingly easier to meet one of them, it becomes increasingly harder to fulfill the other and vice versa. This conflict is, at its core, a grandparent–grandoffspring conflict analogous to parent–offspring conflicts (Trivers, 1974). The result of this is that there is a narrow range over which we should expect grandmothering to evolve and then to persist. In other words, we should in fact expect grandmothering to be rare.

The rest of this study is organized as follows: In the next section, we lay out our model and assumptions. Following this, we explicitly calculate the expected inclusive fitness for the two different strategies. We then find the evolutionary stability threshold, noting that if grandmothering is to be immune to evolutionary cheating, the regular grandmothering strategy should have a higher fitness. In the proceeding section, we derive the eligibility threshold. We then use ancestral parameter values to calculate explicitly these thresholds, demonstrating the conflict between the two. Finally, we summarize our findings and suggest potential tests for the Grandmother Hypothesis.

## 2 Model

As elsewhere (Kim et al., 2014), we assume that individuals transition through six possible life‐history stages: unweaned, weaned, independent, fertile, postfertile, and frail. If we denote the age of an individual by *x*, we can write these life stages as unweaned $x\in [0,\tau_{1})$, weaned $x\in [\tau_{1},\tau_{2})$, independent $x\in [\tau_{2},\tau_{3})$, fertile $x\in [\tau_{3},\tau_{4})$, postfertile $x\in [\tau_{4},\tau_{5})$, and frail $x\in [\tau_{5},d]$, where *d* is some maximum expected life span.

Once individuals reach the postfertile period, we assume that they provide care for some of their grandchildren. We denote the number of fertile children an individual has by κ and the number of grandchildren a postfertile individual can care for by α. As infants are highly dependent on their mothers initially (for example, on their milk in the case of mammals), we further assume that intergenerational care can only be given once any given grandchild is weaned ($x>\tau_{1}$).

We will eventually compare the fitness of individuals that provide grandmothering as outlined above with others that instead continue to reproduce themselves. Such evolutionary cheaters will have an older age where their postfertile period starts. We denote this age by $\tau_{4m}$. In this case, the later life stages will be given by fertile $x\in [\tau_{3},\tau_{4m})$, postfertile $x\in [\tau_{4m},\tau_{5})$, frail $x\in [\tau_{5},d]$. Finally, we define the average time to conception and gestation by β.

## 3 Fitness

If it occurs that individuals with a shorter postfertile phase achieve a higher fitness, we should expect selection to act on the shortening of this stage, reducing it further. In such a scenario, the postfertile stage and hence the ability to grandmother should disappear.

As the only difference in the two strategies occurs during one stage, to compare them, it is sufficient to compare their inclusive fitnesses over that stage. In particular, we focus attention on the period defined by

(1) $$\tau_{4m}-\tau_{4}.$$

In the absence of grandmothering, an individual will have to raise their own infants to $\tau_{2}$ (independence). As the average time to conception and gestation is β, over our period of interest, an individual will be able to produce

(2) $$\frac{\tau_{4m}-\tau_{4}}{\beta +\tau_{2}}$$

infants.

Similarly, their κ fertile children will be able to produce the same amount. Thus, the inclusive fitness (r_m_) over that period of an individual without grandmothering will be

(3) $$r_{m}=\frac{1}{2}\left(\frac{\tau_{4m}-\tau_{4}}{\beta +\tau_{2}}\right)+\frac{\kappa }{4}\left(\frac{\tau_{4m}-\tau_{4}}{\beta +\tau_{2}}\right),$$

where we have added the appropriate relatedness coefficients to distinguish children and grandchildren.

In the alternative scenario, an individual does not produce any infants themselves over our period of interest. Instead, they provide care for α of their grandchildren, allowing α of their own children to reproduce earlier than $\tau_{2}$ at age $\tau_{1}$. The remainder of their children (if there are any) will, however, have to raise their infants to $\tau_{2}$. Hence, the inclusive fitness (r) of an individual that grandmothers as usual will be

(4) $$r=\frac{1}{4}\left(\alpha \left(\frac{\tau_{4m}-\tau_{4}}{\beta +\tau_{1}}\right)+\left(\kappa -\alpha \right)\left(\frac{\tau_{4m}-\tau_{4}}{\beta +\tau_{2}}\right)\right),$$

where again, the weight, 1/4, accounts for relatedness.

## 4 Evolutionary Stability Threshold

For grandmothering to be an ESS and immune to evolutionary cheating (Maynard Smith, 1982), it must be that the fitness benefits of providing intergenerational care outweigh the costs of not continuing one's own reproduction. In other words, it must be that the fitness of the regular grandmothering strategy is higher than the strategy with a reduced postfertile stage so that

(5) $$r>r_{m}.$$

Using (3) and (4) and rearranging, we find that

(6) $$\alpha >2\left(\frac{\beta +\tau_{1}}{\tau_{2}-\tau_{1}}\right).$$

There is hence a threshold value of number of grandchildren that must be cared for if grandmothering is to be maintained in a population. Further, this threshold depends crucially on the ratio of the time it takes to produce and wean an infant and the duration of the weaned stage. For example, for this threshold to be less than one, the average weaned stage of a species must be at least two times as long as the stage previous. Whether or not this condition is met depends on the ecology, physiology, and developmental rate of a species. It is important to note that this condition says nothing about how grandmothering may have initially arose, simply when it will persist. In this way, it does not suggest that grandmothering evolved by giving up reproduction.

## 5 Life‐History Threshold

If a grandmother is to care successfully for at least one set of grandchildren, they must live long enough. More precisely, it should be that the expected postfertile period is longer than the weaned period so that

(7) $$\tau_{5}-\tau_{4}>\tau_{2}-\tau_{1}.$$

If the postfertile period were less than the weaned period, the grandmother would die before any of the infants they are caring for reach independence, resulting in their likely death too. In this case, a grandmothering strategy cannot provide any evolutionary advantage. Given how highly dependent all human infants are, even postweaning, we do not think this assumption is unreasonable. See (Sear & Mace, 2008), for the largest review of its kind on infant mortality and kin help.

It is important to note that this eligibility threshold is in opposition to the stability threshold. The stability threshold becomes increasingly easier to meet as the weaned period $\tau_{2}-\tau_{1}$ increases. However, an increase in the same stage makes the eligibility threshold more difficult to achieve. While it is in the interest of the infant to have an increasingly higher $\tau_{2}$, grandmothers will spread more of their genes if this age is lower. In other words, there exists a grandparent–grandoffspring conflict entirely akin to parent–offspring conflicts (Trivers, 1974). We suggest that this conflict goes some way toward explaining the rarity of grandmothering.

## 6 Ancestral Parameter Estimates

In the case of ancestral humans, a previous study has estimated β to be approximately 1 year (Kim et al., 2014). This is found by assuming an average time to conception of half a year which is added to an average taken over the gestation times of humans, gorillas, and chimpanzees. Age of weaning, $\tau_{1}$, is taken to be 2 years. This is based on the observation that in some human populations, after this age, a mother's death does not increase offspring mortality (Sear & Mace, 2008). Additionally, it has been noted that chimpanzees can survive the death of their mother at this age if (a rare event) they are adopted (Mace, 2000).

**Figure 1:**
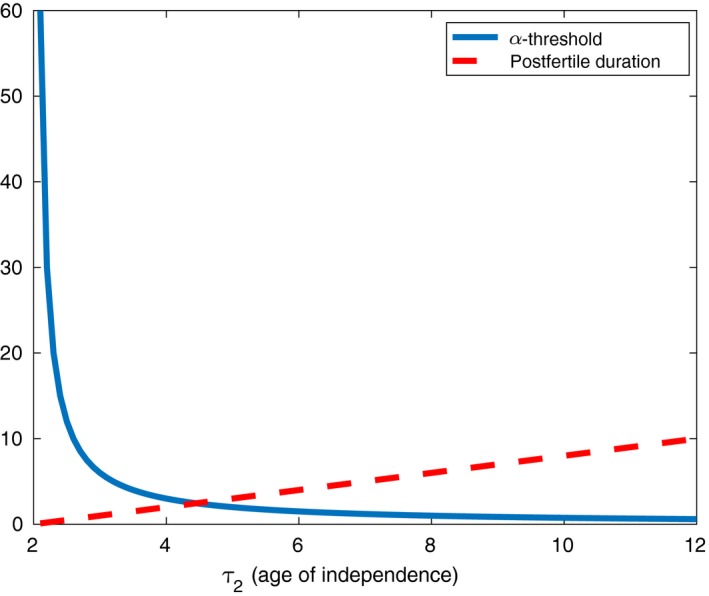
Minimum number of infants a grandmother must provide care for (solid line) and minimum postfertile duration (dotted line) as a function of age of independence. Both must be met for grandmothering to persist. Here, $\tau_{1}=2$ and β=1

With these values, we can explicitly calculate the stability threshold (solid line) for any value of $\tau_{2}$ as in Figure 1. Observe that as $\tau_{2}$ increases, grandmothers must care for *significantly* fewer infants for grandmothering to provide evolutionary benefits. The eligibility threshold (dotted line) is also plotted in Figure 1 for the same ancestral parameter estimates. In contrast to the stability threshold, it increases as a function of $\tau_{2}$.

Notice that, in Figure 1, in the region to the right of $\tau_{2}=8$, a grandmother must only care for one infant for grandmothering to be an ESS. However, here the postfertile period must be in excess of 6 years. By contrast, to the left where the postfertile period can be shorter, a grandmother must care for more than one. This might be fulfilled if, as the Grandmother Hypothesis suggests, the environment of our ancestors changed. Further left still however we see that the stability threshold eventually becomes biologically unrealistic. Only through an intermediate range are both thresholds biologically realizable.

## 7 Discussion

Intergenerational care, via the Grandmother Hypothesis, has been implicated in the evolution of postfertile human longevity (Hawkes, 2003; Hawkes et al., 1998; Kim et al., 2012, 2014). The extension of lifespan without an extension of fertility may be evolutionarily beneficial if, by caring for grandchildren, the interbirth intervals of one's children are shortened. A valid objection to this hypothesis, however, is if grandmothering does allow an organism to spread more of its genes, why is it so rare? Here, we developed a simple quantitative model to investigate this objection.

This model assumed that individuals transition through six possible life‐history stages and that there is an average time to conception and of gestation. With this setup, we noted that if a grandmother strategy is to allow the provision of enough care, the postfertile stage should be longer than the weaned staged of their grandchildren. Indeed, it is also in the interest of children, who have twice the amount of genetic material at stake, for this to be the case. This led to a simple eligibility threshold.

We additionally asked the question, assuming grandmothering has evolved (by whichever route), when will it persist. Competing with individuals that continue reproducing into old age, we found that grandmothering will achieve a higher fitness only if care can be provided for a threshold number of grandchildren. This stability threshold depended on the ratio of the time it takes to produce and wean an infant and the duration of the weaned stage.

For grandmothering to evolve in the first place, and then for it to persist, both thresholds need to be met. Crucially, however, there is conflict between these two conditions. As it becomes increasingly easier to meet one, it becomes increasingly more difficult to meet the other.

Taking ancestral parameter estimates available in the literature (Kim et al., 2014), we then explicitly calculated both thresholds as functions of the age where individuals become independent. This highlighted that, for our ancestors, there was a small window of opportunity for grandmothering to evolve and persist. Our analyses have hence shown that in fact one should instead *expect* grandmothering to evolve infrequently.

This window, if the Grandmother Hypothesis is correct, was realized because of a fortuitous intersection of ecology and phylogeny. In particular, the Grandmother Hypothesis suggests that savanna‐like environments, which increased during the Pliocene epoch, led our ancestors to subsist on plant foods that were manageable by older and bigger individuals but not by juveniles (Hawkes & Blurton Jones, 2005). This may have allowed the stability threshold to be met, particularly with economies of scale arising from grandchildren approaching independence. The eligibility threshold, if they live long enough, is also met by our closest relatives the chimpanzees (Cohen, 2004; Robson & Wood, 2008). However, chimpanzees that do have postfertile periods are not the norm but the exception; overall postreproductive representation is low (Levitis et al., 2013). Nonetheless, it is possible that our last common ancestor also fulfilled this criterion. In this case, the question of “why us and not them?” can be answered by ecology and in particular the stability threshold we derived.

Unlike previous modeling on this topic, the simple thresholds of this study all involve life‐history traits that can be measured. In this way, our work aims to make the evolutionary view of grandmothering testable. If grandmothering is observed and these conditions are not met, it would suggest that grandmothering is occurring for different reasons. This would then in turn cast doubt on the link between intergenerational care and postfertile longevity. Further, the results of this study suggest particular scenarios where we might search for nonhuman animals that grandmother. Additionally, once data are available, these thresholds could be used to see whether the Grandmother Hypothesis can apply to other organisms (such as pilot and killer whales) that we know to have significant postreproductive representation (and not simply postreproductive viability, which is often an artifact of captivity; Levitis et al., 2013).

At the heart, our results are a grandparent–grandoffspring conflict that results in difficulty in fulfilling both necessary thresholds. While the literature on parent–offspring conflicts is prolific, formal work on intergenerational conflicts appears comparatively scant. In future work, it will be interesting to fully tease out the ramifications of such a conflict.

## Conflict of Interest

None declared.

## Authors’ Contributions

JMF carried out the research. JMF and MBB wrote the manuscript.
